# Bulk gDNA Sequencing of Antibody Heavy-Chain Gene Rearrangements for Detection and Analysis of B-Cell Clone Distribution: A Method by the AIRR Community

**DOI:** 10.1007/978-1-0716-2115-8_18

**Published:** 2022

**Authors:** Aaron M Rosenfeld, Wenzhao Meng, Kalisse I Horne, Elaine C Chen, Davide Bagnara, Ulrik Stervbo, Eline T Luning Prak

**Affiliations:** 1 Department of Pathology and Laboratory Medicine, Perelman School of Medicine, University of Pennsylvania, Philadelphia, PA, USA; 2 Department of Pathology Microbiology and Immunology, Vanderbilt University Medical Center, Nashville, TN, USA; 3 Department of Experimental Medicine, University of Genoa, Genoa, Italy; 4 Center for Translational Medicine and Immune Diagnostics Laboratory, Medical Department I, Marien Hospital Herne, University Hospital of the Ruhr-University Bochum, Herne, Germany

**Keywords:** Antibody, Clone, Lineage, Immune repertoire profiling, Immunoglobulin, V(D)J recombination, Next-generation sequencing

## Abstract

In this method we illustrate how to amplify, sequence, and analyze antibody/immunoglobulin (IG) heavy-chain gene rearrangements from genomic DNA that is derived from bulk populations of cells by next-generation sequencing (NGS). We focus on human source material and illustrate how bulk gDNA-based sequencing can be used to examine clonal architecture and networks in different samples that are sequenced from the same individual. Although bulk gDNA-based sequencing can be performed on both IG heavy (IGH) or kappa/lambda light (IGK/IGL) chains, we focus here on IGH gene rearrangements because IG heavy chains are more diverse, tend to harbor higher levels of somatic hypermutations (SHM), and are more reliable for clone identification and tracking. We also provide a procedure, including code, and detailed instructions for processing and annotation of the NGS data. From these data we show how to identify expanded clones, visualize the overall clonal landscape, and track clonal lineages in different samples from the same individual. This method has a broad range of applications, including the identification and monitoring of expanded clones, the analysis of blood and tissue-based clonal networks, and the study of immune responses including clonal evolution.

## 1 Introduction

Antibodies or immunoglobulins (IGs) on B cells are generated through somatic recombination of variable (V), diversity (D), and joining (J) genes [1, 2] and further diversified through somatic hypermutation (SHM) [3, 4]. The collection of different B cells in an individual, also known as the immune repertoire, is complex, containing many different B cells with different antibodies. B cells that derive from the same progenitor are clonally related and harbor gene rearrangements that are identical or have very similar nucleotide sequences (differing only by SHM or sequencing errors). The grouping of antibody gene rearrangement sequences into clones provides a means of characterizing the immune repertoire with respect to the distribution, size, complexity, and dynamics of clones in different cell types and tissues [5–7].

Here we describe a homebrew method, with primer sequences adapted for NGS from the BIOMED2 IG heavy-chain (IGH) PCR assays [8], to evaluate samples for evidence of B-cell clonal expansion and track clones in bulk gDNA samples. Similar methods exist as commercial services (e.g., Adaptive Biotechnologies, iRepertoire), and there are also similar homebrew methods for the analysis of T-cell AIRR-seq data (e.g., [9]). This homebrew method for IGH rearrangements uses multiplex PCR and can be scaled to very high cell inputs as described in [10]. DNA is more robust than RNA and has a simpler relationship to cell numbers (one template per cell) than RNA. For these reasons, bulk gDNA-based sequencing is typically the method of choice for clinical-grade assays to evaluate malignant clonal expansions [11], as well as the in-depth study of clones in different tissues to study clonal networks in the body [10]. The method shown uses long reads that are adequate for robust IGHV gene alignment and evaluation of SHM, but this method can also be performed with shorter reads, depending upon the sample type and DNA quality.

In this chapter, we also illustrate how to use pRESTO [12] and ImmuneDB [13] to analyze sequencing data generated following the wet bench protocol. In this dry bench analysis, we describe how to filter the raw read data, group highly similar rearrangements into clones using both the IGHV gene and CDR3 sequences, estimate clone size distributions, and track clones of interest in other samples.

## 2 Materials

### 2.1 Primers

All IG gene amplification primers are synthesized by Integrated DNA Technologies, and HPLC purification is recommended for sequences that are longer than 60 bp and any sequence that contains one or more “Ns” (random nucleotides). Dual indices are provided to distinguish clone identification from tracking primers (*see* Note 1).

1. Human (Hu) IGH amplification primers for clone identification:
  NexteraR2-Hu-VH1-FW1:GTCTCGTGGGCTCGGAGATGTGTATAAGAGACAGGGCCTCAGTGAAGGTCTCCTGCAAG
  NexteraR2-Hu-VH2-FW1:GTCTCGTGGGCTCGGAGATTGTATAAGAGACAGGTCTGGTCCTACGCTGGTGAAACCC
  NexteraR2-Hu-VH3-FW1:GTCTCGTGGGCTCGGAGATGTGTATAAGAGACAGCTGGGGGGTCCCTGAGACTCTCCTG
  NexteraR2-Hu-VH4-FW1:GTCTCGTGGGCTCGGAGATGTGTATAAGAGACAGCTTCGGAGACCCTGTCCCTCACCTG
  NexteraR2-Hu-VH5-FW1:GTCTCGTGGGCTCGGAGATGTGTATAAGAGACAGCGGGGAGTCTCTGAAGATCTCCTGT
  NexteraR2-Hu-VH6-FW1:GTCTCGTGGGCTCGGAGATGTGTATAAGAGACAGTCGCAGACCCTCTCACTCACCTGTG
  NexteraR1-Hu-JHmix1:TCGTCGGCAGCGTCAGATGTGTATAAGAGACAGTACGTNCTTACCTGAGGAGACGGTGACC
  NexteraR1-Hu-JHmix2:TCGTCGGCAGCGTCAGATGTG TATAAGAGACAGCTGCNCTTACCTGAGGAGACGGTGACC
  NexteraR1-Hu-JHmix3:TCGTCGGCAGCGTCAGATGTG TATAAGAGACAGAGNCTTACCTGAGGAGACGGTGACC
2. Hu IGH amplification for clone tracking:
  These primers use dual ID barcodes to distinguish them from the identification sample amplicons (the bold font indicates the barcode sequences).
  NexteraR2-Barcoded-Hu-VH1-FW1: GTCTCGTGGGCTCGGAGATGTGTATAAGAGACAG**AGGCTATA**GGCCTCAGTGAAGGTCTCCTGCAAG
  NexteraR2-Barcoded-Hu-VH2-FW1: GTCTCGTGGGCTCGGAGATGTGTATAAGAGACAG**GCCTCTAT**GTCTGGTCCTACGCTGGTGAAACCC
  NexteraR2-Barcoded-Hu-VH3-FW1: GTCTCGTGGGCTCGGAGATGTGTATAAGAGACAG**AGGATAGG**CTGGGGGGTCCCTGAGACTCTCCTG
  NexteraR2-Barcoded-Hu-VH4-FW1: GTCTCGTGGGCTCGGAGATGTGTATAAGAGACAG**TCAGAGCC**CTTCGGAGACCCTGTCCCTCACCTG
  NexteraR2-Barcoded-Hu-VH5-FW1: GTCTCGTGGGCTCGGAGATGTGTATAAGAGAC AG**CTTCGCCT**CGGGGAGTCTCTGAAGATCTCCTGT
  NexteraR2-Barcoded-Hu-VH6-FW1: GTCTCGTGGGCTCGGAGATGTGTATAAGAGACAG**TAAGATTA**TCGCAGACCCTCTCACTCACCTGTG
  NexteraR1-Barcoded-Hu-JHmix4: TCGTCGGCAGCGTCAGATGTGTATAAGAGACAG**ATTACTCG**TACGTNCTTACCTGAGGAGACGGTGACC
  NexteraR1-Barcoded-Hu-JHmix5: TCGTCGGCAGCGTCAGATGTGTATAAGAGACAG **TCCGGAGA**CTGCNCTTACCTGAGGAGACGGTGACC
  NexteraR1-Barcoded-Hu-JHmix6: TCGTCGGCAGCGTCAGATGTGTATAAGAGACAG**CGCTCATT**AGNCTTACCTGAGGAGACGGTGACC
3. NexteraXT index primers S5XX and N7XX. These primers are synthesized with HPLC purification to create sets A, B, C, and D for different barcode combinations (available from Illumina).

### 2.2 DNA Extraction

Use molecular biology-grade reagents.

1. Isopropanol, DNase/RNase free.
2. 200 proof ethanol.
3. 10 mM Tris and 0.1 mM EDTA, pH 8.0.
4. DNase/RNase-free water.
5. Glycogen.
6. RBC lysis solution (Qiagen).
7. Cell lysis solution (Qiagen).
8. Protein precipitation solution (Qiagen).
9. RNase A solution (Qiagen).
10. DNA-off (Thermo Fisher Scientific).

### 2.3 Library Preparation

1. Multiplex PCR Kit (Qiagen).
2. Ultrapure agarose (Thermo Fisher Scientific).
3. 100 bp DNA Ladder (New England Biolabs).
4. 50× Tris-acetate electrophoresis buffer (Quality Biological)
5. Agencourt AMPure XP beads (Beckman Coulter).
6. Gel Extraction Kit (Qiagen).
7. 3 M sodium acetate, pH 5.5 (Sigma).
8. 10 mg/ml ethidium bromide aqueous solution (Sigma-Aldrich).

### 2.4 Library QC and Sequencing

Use molecular biology-grade solutions.

1. 10 M Sodium hydroxide solution, BioUltra (Sigma-Aldrich).
2. 600-Cycle MiSeq Reagent Kit v3 (Illumina).
3. Qubit dsDNA High-Sensitivity Kit (EMSCO/Thermo Fisher Scientific).
4. KAPA Library Quantification Kit (EMSCO/Thermo Fisher Scientific).
5. PhiX Control V3 Kit (Illumina).
6. Tween 20 (Sigma-Aldrich).

### 2.5 Disposables

1. 96-well ABI-style PCR plate (Thomas Scientific).
2. Plate-sealing film, aluminum, cold storage, and sterile (Thomas Scientific).
3. Microseal “B” adhesive seals for thermo cycling (Bio-Rad Laboratories).
4. 1.5 ml Posi-Click tubes (Thomas Scientific).
5. Reagent reservoir, 25 ml, sterile, and individually wrapped (Thomas Scientific).
6. DNA LoBind tubes, 1.5 ml (Eppendorf).
7. PCR plate 96 LoBind, semi-skirted (Eppendorf).
8. Qubit assay tubes (Life Technologies).
9. Polypropylene conical tube 15 ml bulk wrap sterile (Thomas Scientific).
10. P2/P10 extra-long filter pipet tips (Thomas Scientific).
11. P-20 filter pipet tips (Thomas Scientific, *see* Note 2).
12. P-200 filter pipet tips (Thomas Scientific).
13. P-1000E filter pipet tips (Thomas Scientific).

### 2.6 Equipment

1. Veriti 96-well thermal cycler.
2. NanoDrop 1000 spectrophotometer.
3. Agilent 2100 bioanalyzer.
4. Qubit 4 fluorometer.
5. Illumina MiSeq.
6. PCR workstation (C.B.S. Scientific).
7. 96S super ring magnet plate (Thomas Scientific).
8. Labnet mini plate spinner (Thomas Scientific).
9. Gel Doc XR Imaging System with Universal Hood II (Bio-Rad).
10. Owl™ EasyCast™ B2 mini gel electrophoresis system.

## 3 Methods

The major steps of the wet bench procedure are outlined in Fig. 1.

### 3.1 Lab Setup

The lab should have separate areas for pre-PCR and post-PCR work, to prevent contamination. Two separate rooms are recommended with all DNA extraction: the PCR setup is performed in the pre-PCR room, and all of the gel running, AMPure bead purification, and sequencing are performed in the post-PCR room. Use DNA-off to wipe down the workstation and UV treat pipets before and after each experiment.

### 3.2 DNA Purification

This protocol starts from high-purity genomic DNA (gDNA) that has been isolated from a population of cells, such as peripheral blood mononuclear cells (PBMCs), or cells from tissues or sorted cells (Fig. 1a and *see* Note 3). To prepare the sorted cells for sequencing, sort cells directly into 300 μl of cell lysis solution if the expected lymphocyte yield is less than 50,000 cells and use a DNA LoBind tube. If the expected yield is more than 50,000 cells per population, sort the cells into sorting buffer, centrifuge the cells, remove the supernatant, and resuspend the cell pellet in a cell lysis buffer (add 300 μl cell lysis buffer for up to two million cells). DNA is extracted from whole blood, bone marrow, or sorted cells using protocols from Gentra Puregene (Qiagen) handbook using the manufacturer’s recommendations. Outlined below is the protocol (with notes) for 3 ml of whole blood.

1. Mix 3 ml whole blood with 9 ml of RBC lysis solution in a 15 ml conical tube, and gently invert ten times. Incubate at room temperature for 5 min (minutes), and invert the mixture at least once during the incubation (*see* Note 4).
2. Centrifuge at 2000 × *g* for 2 min to pellet the white blood cells. Carefully discard the supernatant by pipetting or pouring the supernatant to a waste tank containing water with 10% bleach. Leave behind ~200 μl of the residual liquid, and vortex to resuspend the pellet in the residual liquid (*see* Note 5).
3. Add 3 ml of cell lysis solution, and vortex for 10 s (seconds). Stopping point: Once samples are fully suspended in cell lysis solution, the DNA will be stable for 2 years (*see* Notes 6 and 7).
4. Add 1 ml protein precipitation solution, and vortex for 20 s at high speed. Centrifuge at 2000 × *g* for 5 min (*see* Note 8).
5. Transfer the supernatant to a new 15 ml conical tube, and add 3 ml isopropanol. Mix by inverting 30 times until the DNA is visible as threads or a clump (*see* Note 9). Stopping point: DNA can be precipitated overnight.
6. Centrifuge at 2000 × *g* for 3 min. After centrifugation, the DNA may be visible as a small white pellet. Carefully pour off the supernatant to a waste isopropanol/ethanol container. Drain the residual liquid in the tube by inverting on a clean piece of absorbent paper.
7. Add 3 ml of 70% ethanol (prepared with molecular biology grade ethanol and DNase/RNase-free water), and invert several times to wash the DNA pellet. Centrifuge at 2000 × *g* for 1 min, and carefully pour the supernatant to a waste isopropanol/ethanol container. As the DNA pellet may be loose at this step, decant the liquid from the tube carefully.
8. Perform a quick spin at 2000 × *g* for 30 s to bring down the residual ethanol to the bottom of the tube, and use a P-200 μl filter tip to remove the residual ethanol. Allow the DNA pellet to air dry for 10 min or until no ethanol can be observed. Note: Avoid overdrying the DNA pellet, as then the DNA will be difficult to dissolve.
9. Resuspend DNA in 100 ul of TE (low EDTA) buffer (10 mM Tris and 0.1 mM EDTA), and check the DNA quality using NanoDrop. The OD_260_/OD_280_ ratio should be close to 1.8. If the DNA concentration is <100 ng/μl using the NanoDrop instrument, repeat the DNA concentration measurement using Qubit HS DNA Kit for a more accurate measurement.

### 3.3 Template Amplification and Initial Quality Control

Before beginning, make sure that all of the workstations are clean, and perform all template amplification procedures in a separate pre-PCR area. Aliquot all primers (equimolar mixture of primers for both VH and JH primer mixes), PCR-grade water, and PCR master mix buffers before use. The PCR product that is amplified from gDNA is shown in Fig. 1b.

1. Use water and PCR master mix from Qiagen Multiplex PCR Kit, and prepare the PCR mix (*see* Notes 10–13):

**Table:** 

Reagent	Volume (μl)

DNA	4
2× master mix PCR buffer	12.5
5′ VHFW1 mix (5 μM)	3
3′ JH mix (5 μM)	3
Nuclease-free water	2.5
**Total volume**	**25**
2. Thermal cycling. If using plates, use microseal B adhesive seal. Perform a quick spin of the plate before loading onto the thermal cycler, and run the following program:
  *First PCR program*.

**Table:** 

Temperature and time	Number of cycles

95 °C 7 min	1
95 °C 45 s, 60 °C 45 s, 72 °C 90 s	35
72 °C 10 min	1
  Stopping point: Amplified samples can be stored at 4 °C for up to 48 h.
3. Agarose gel electrophoresis of PCR products (*see* Note 14). Gel electrophoresis is performed to ensure that the first-round PCR has generated a sufficient quantity of amplicons of the correct length and that there is no evidence of contamination in the negative controls.
  - Prepare a 2% agarose gel (ultrapure agarose) in 1× TAE buffer. Ethidium bromide can be mixed into the gel, or the gel can be stained afterward to visualize the DNA.
  - Load 5 μl of the first-round PCR amplicons per lane, and check the amplicon size under UV light by comparing the products to the molecular weight ladder (100 bp ladder).
  - The amplicon on the gel should be the same as the positive control band, and the size is ~440 bp. If water and fibroblast DNA controls show contamination, the whole experiment needs to be rerun.
4. AMPure bead purification (Fig. 1c) is performed on the remaining 20 μl of the sample from the first-round PCR to enrich for PCR products of the appropriate length and remove primers and primer dimers following the manufacturer’s protocol (Beckman Coulter). Aliquot beads before use. Equilibrate beads to room temperature, and prepare fresh 85% ethanol each time before use. Use filter tips.
  - Mix an equal volume of AMPure beads with amplicons (in this case, 20 μl of beads, *see* Note 15).
  - Mix the beads and amplicons together by pipetting up and down 20 times. Incubate the mixture at room temperature for 1 min. Set the plate on the magnet for 5 min until the mixture is clear.
  - Keep the PCR plate on the magnet, remove the supernatant, and discard.
  - Wash the beads by adding 180 μl of fresh 85% ethanol, *do not mix*, incubate at room temperature for 30 s, and remove and discard the supernatant.
  - Use P2/P10 extra-long tips to remove the residual ethanol from each well, and air dry at room temperature for up to 5 min. Note: Do not allow beads to air dry for more than 5 min.
  - Remove the PCR plate from the magnet, add 40 μl of TE (low EDTA) buffer into each sample well. Mix by pipetting up and down ten times to resuspend the beads. Incubate at room temperature for 2 min.
  - Return the PCR plate to the magnet, and incubate at room temperature for 5 min. With the plate on the magnet, transfer 38 μl of the eluates to a new 96-well PCR plate. Stopping point: At this step, the new plate with the purified first PCR amplicons can be sealed and stored at −20 °C for later use.

### 3.4 Second-Round PCR and Product Purification

In this section of the protocol, the bead-purified amplicons from the first step are amplified using primers that are tagged with Illumina barcodes. A schematic illustration of the PCR product is shown in Fig. 1d. All procedures for preparing the PCR mix are performed in the pre-PCR room, *except* for the addition of the first-round PCR amplicons, which is performed in a PCR hood in the post-PCR room. Aliquot all primers (Nextera XT index primers), PCR-grade water, and PCR master mix buffers before use.

1. Use water and PCR master mix from the Qiagen Multiplex PCR Kit, and prepare the PCR mix:

**Table:** 

Reagent	Volume (μl)

Purified first-round PCR amplicons	4
2× master mix PCR buffer	12.5
NexteraXT index primer S5XX	2.5
NexteraXT index primer N7XX	2.5
Nuclease-free water	3.5
**Total volume**	**25**
2. Run the second-round PCR program:

**Table:** 

Temperature and time	Cycles

95 °C 10 min	1
95°C 30 s, 60°C 30 s, 72°C 45 s	8
72°C 10 min	1
  Stopping point: Amplified samples can be stored at 4 °C for up to 48 h.
3. Sample pooling and analysis of pooled second-round PCR products (*see* Note 16).
  - Add equal volumes (typically ~5 μl) of the individual sample amplicons (replicates) together into a “pooled library” for sequencing. Samples can be pooled together at this stage, because the amplicons have sample-specific barcodes.
  - Prepare a 2% agarose gel, and add 5 μl of the second-round PCR amplification mixture. The expected amplicon size on the gel is ~510 bp and should be present in the positive control sample. If water or fibroblast have amplification products, the second-round PCR experiment needs to be rerun. Stopping point: The second-round PCR samples can be stored at −20 °C in a post-PCR freezer for later use (*see* Note 17).
4. Optional gel extraction step. If primer dimers are observed at the size of ~200 bp, a gel purification step using QIAquick Gel Extraction Kit is recommended to enrich for products of the right length for sequencing. Gel extraction is preferred over AMPure beads for this step, because the beads do not remove this amplicon size well.
  - Run the pooled samples on a 2% agarose gel with a low-voltage setting (~60 V) to allow the amplicons to migrate slowly on the gel.
  - After 3 h of gel running, cut out the expected size (510 bp) band under long wavelength UV light to minimize DNA damage. Weigh the gel slice in a 1.5 ml Eppendorf tube.
  - Add 3 volumes of buffer QG to 1 volume of gel (100 mg gel corresponds to ~100 μl of liquid volume). The maximum amount of gel per spin column is 400 mg. Incubate at 50 °C for 10 min (invert the tube to help dissolve gel) or until the gel slice has dissolved completely.
  - If the color of the mixture is orange or violet, add 10 μl of 3 M sodium acetate until the color turns yellow. Add 1 gel volume of isopropanol to the sample, and mix by inverting the tube ten times.
  - Apply 750 μl of the gel-isopropanol mixture to a QIAquick spin column in the provided 2 ml collection tube, and centrifuge at 17,900 × *g* for 1 min.
  - Discard the flow-through, and place the QIAquick column back into the same tube.
  - Apply the rest of the mixture (if any is remaining) to the same column, and repeat steps 4e and 4 f.
  - Add 750 μl buffer PE to the QIAquick column, and centrifuge at 17,900 × *g* for 1 min to wash the column. Discard flow-through, and place the QIAquick column back into the same collection tube.
  - Centrifuge the QIAquick column for 1 min to remove the residual wash buffer, and place the QIAquick column into a clean 1.5 ml Eppendorf tube.
  - Add 50 μl buffer EB to the center of the QIAquick membrane, let the column stand for 2–3 min, and then centrifuge for 1 min. Stopping point: Gel-purified product (the eluate in the clean 1.5 ml Eppendorf tube) can be stored at −20 °C in the post-PCR freezer for later use.

### 3.5 Library Pooling, Purification, and Quantification

1. Gather up all of the pooled libraries and gel-purified pooled libraries, if any, that are going to be included in the sequencing run (*see* Note 18).
2. Starting from the pooled libraries, perform two rounds of AMPure bead purification as described previously (Fig. 1e).
3. The final purified libraries can be eluted in ½ or ¼ of the original pooled sample volume to concentrate the library, if needed.
4. Run 1 μl of each pooled library with a Bioanalyzer high-sensitivity DNA assay to verify the size and purity. Check the concentration of each pooled library on Qubit using a dsDNA High-Sensitivity Kit with 2 μl of each pool.
5. Once the molarity is calculated for each pooled library (*see* Note 19), normalize the library inputs in the sequencing run. The goal is to have the number of molecules per sample be equal across the different libraries. For example, suppose that one pooled library (library A) has 34 samples with an overall molarity of 50 nM and a second pooled library (library B) has 46 samples with a molarity of 35 nM. If 10 μl of library B is used for the final pooled library, then the volume of library A is calculated by solving for A in the following expression:
$$\begin{array}{l} (\text{A}\;\text{μl}\times 50\;\text{nM})/34\;\text{samples}=(10\;\text{μl}\times 35\;\text{nM})/46\;\text{samples}.\\ \text{A}=5.17\;\text{μl}. \end{array}$$
  The concentration of the final pooled library is determined by Qubit and calculated as molarity (*see* Note 19).

### 3.6 Sequencing

1. Prepare a fresh dilution of 0.2 N NaOH. Dilute the original 10 N NaOH to 1 N, and discard the aliquot after 3 months. Mix 80 μl of water and 20 μl of 1 N NaOH for a total of 100 μl of 0.2 N NaOH.
2. Prepare 4 nM of the final pooled sequencing library by diluting the concentrated one with TE (low EDTA).
3. Mix 5 μl of 0.2 N NaOH and 5 ul of 4 nM library by pipetting up and down for 20 times in a 1.5 DNA LoBind tube. Denature at room temperature for 5 min.
4. Add 990 μl prechilled HT1 (from the MiSeq Kit), and incubate on ice immediately. The final concentration for the denatured library is 20 pM.
5. Prepare 20 pM of PhiX. Mix 2 μl of PhiX control with 3 μl of TE (low EDTA) in a 1.5 ml DNA LoBind tube by pipetting. Add 5 μl of freshly diluted 0.2 N NaOH, mix by pipetting up and down 20 times, and incubate at room temperature for 5 min. Next, add 990 μl prechilled HT1 (from the MiSeq Kit), and incubate on ice immediately (*see* Note 20).
6. To spike in 10% PhiX into the final sequencing library, take 100 μl of the 20 pM denatured library out and discard, and add in 100 μl of 20 pM denatured PhiX. This will yield 20 pM of the final sequencing library with 10% PhiX (*see* Note 21). Load 600 μl of this library to the pre-thawed MiSeq cartridge MiSeq^®^ Reagent Kit v3 (2X300 cycles). The run takes 2.5 days to complete.
7. General sequencing run QC. For the MiSeq (2X300 cycle) V3 Kit, the optimal raw cluster density is 1200–1400 K/mm^2^ (Illumina provides additional details on clustering density online). The percentage of reads for the entire run that have Q scores above 30 (Q30, 1 in 1000 base calls may be incorrect) should be at least 70%. Finally, the percentage of clusters passing filter (PF%) should be > = 80%. If a run does not pass all three of these thresholds, the sequencing should be repeated. Under passing conditions, each replicate has on average 100,000 to 300,000 valid reads (using pRESTO processing with Q30 filtering, please see following sections for data analysis).

### 3.7 Software Installation

Before processing raw sequencing data, analysis software must be installed as follows:

1. Install pRESTO. pRESTO [12] is used for quality control prior to running the rest of the pipeline. It can be installed with pip3 install presto.
2. Install the ImmuneDB Docker image. The ImmuneDB [13] Docker image should be installed for gene identification (via pre-installed IgBLAST), clonal inference, database-backed storage, exporting, and a web interface. For illustrative purposes, we will use version 0.29.10, which can be pulled with docker pull arosenfeld/immunedb:v0.29.10.

### 3.8 Raw Data Processing

Raw data from NGS platforms are generally output in a format providing base calls for each read along with a quality score for each base. Depending on the sequencing method, there are a number of different steps to transform and filter these data into a format that is readily available for further analyses. In general, if reads are paired, the matching 5′ and 3′ reads must be aligned to form full-length sequences. Specifically, each pair of reads is iteratively compared until the maximal number of overlapping nucleotides is found. Nucleotides in the overlapping segment that do not match are assigned the base from whichever read has a higher-quality score.

Following this, short and low-quality sequences should be removed as they do not provide sufficient information to make accurate gene calls. Then, primer sequences which were incorporated into the DNA/RNA templates should be masked as not to skew later mutation analyses. Individual base calls with low confidence (generally either a Phred score < 20 or < 30) should be masked to reduce their influence on downstream analyses. Finally, genes should be annotated with IgBLAST for downstream processing. The commands for this entire process, assuming paired input files from an Illumina-based sequencing platform and applying a Phred quality score filter of 30, are as follows:

1. Locate the sequencing FASTQ files. First, change the working directory to where the sequencing is located. For example, if the data are in $HOME/seq_data, run cd $HOME/seq_data.
2. Run pRESTO:

PairSeq.py −1 *R1*.fastq −2 *R2*.fastq
AssemblePairs.py align −1 *R1*_pair-pass.fastq \
−2 *R2*_pair-pass.fastq \
--coord illumina
FilterSeq.py quality -s *assemble-pass.fastq
FilterSeq.py trimqual -s *quality-pass.fastq -q 30 --win 20
FilterSeq.py length -s *trimqual-pass.fastq -n 100
FilterSeq.py maskqual -s *length-pass.fastq -q 30
FilterSeq.py missing -s *maskqual-pass.fastq -n 10
3. Move the quality-controlled data into a new directory. The remaining steps of this method only use the final resulting files which will end in missing-pass.fastq. These files should now be moved to a location to mount into the ImmuneDB Docker container.

mkdir $HOME/immunedb_share/input
mv *missing-pass.fastq $HOME/immunedb_share/input
4. Annotate raw sequences which have passed general quality control filters with gene information. For IGH sequences V, D, and J genes should be associated with each sequence. IgBLAST is the preferred annotation tool which provides AIRR-compliant output for gene calls in addition to other alignment information [14]. For ease, IgBLAST and a helper script are included in the ImmuneDB Docker image. To begin annotation, perform the following:
  - Run the docker container.
    To begin an interactive session, run the following:

docker run -v $HOME/immunedb_share:/share \
-p 8080:8080 \
-it arosenfeld/immunedb:v0.29.10
    One should see output similar to the following, after which a terminal prompt will be shown:

Moving MySQL to Volume
* Starting MariaDB database server mysqld [ OK ]
Setting up database
Starting webserver
  - Run IgBLAST on the QC’d FASTQ files. In the Docker container, a helper script run_igblast.sh can be used to annotate sequences. Reference genes are provided for humans and mice for IGH, IGL, IGK, TRA, and TRB. In this protocol, we will focus on human IGH. Run the following:

run_igblast.sh human IGH /share/input /share/input
mkdir -p /share/sequences
mv /share/input/*.fast[aq] /share/sequences
    After this step, TSV files annotated in AIRR format [15] will be located in the Docker container at /share/input (which is also accessible at $HOME/immunedb_share/input on the host).

### 3.9 Importing Metadata and Sequence Data into ImmuneDB

1. Specifying sample metadata. Prior to importing these annotated data into ImmuneDB for clonal inference and downstream analyses, metadata must be specified for each sample file.
  - Create a template metadata file. Although a metadata file is simply a TSV which could be created manually, ImmuneDB provides a helper script to create a template as follows:

cd /share/input
immunedb_metadata --use-filenames
  - Add relevant metadata. With the command above, a metadata file with one row per file will be generated, and the sample name for each file will be set to the filename stripped of its extension.
    On the **host**, open the metadata file in a spreadsheet editor. The headers included by default are required; file_name and sample_name will already be filled in from the previous step, but the study_name, subject must be filled in (*see* Note 22).
2. Importing sequences into ImmuneDB. The data are now ready for importing and further processing before clonal inference. To do so, in the Docker container, run the following steps.
  - Create a database for the project. The first step is to create a database into which the AIRR-compliant sequencing data annotated by IgBLAST will be stored. For this method we will call the database my_db, but it can be any valid name for a MySQL database (*see* Note 23).

immunedb_admin create my_db /share/configs
  - Import the annotated data and trace duplicate sequences. The next commands import all the annotated sequences into the previously created database and annotate (collapses) duplicate reads within and between samples. Counting duplicates is useful for downstream filtering and clone size estimation.

immunedb_import /share/configs/my_db.json airr \
/root/germlines/igblast/human/IGHV.gapped.fasta \
/root/germlines/igblast/human/IGHJ.gapped.fasta \
/share/input \
--trim-to 80
immunedb_collapse /share/configs/my_db.json
    One important parameter in the previous commands is --trim-to. This masks the bases on the 5′ end of each read with the ambiguity character *N*. This avoids the primer sequences, which are incorporated into the resulting reads, from being incorporated into downstream mutational analyses. The value of 80 was chosen for this chapter due to the use of framework 1 (FWR1) primers. If different primers are used, the IMGT position of the 3′ end of the primer sequence should be used instead.

### 3.10 Clonal Inference from Sequencing Data and General Statistics

1. Once the data are imported and collapsed, sequences likely originating from a common progenitor cell can be grouped into clones.

immunedb_clones /share/configs/my_db.json cluster
  The default parameters used by immuneDB to specify clonally related sequences are the use of the same IGHV and IGHJ genes, the same CDR3 length, and at least 85% amino acid sequence similarity in the CDR3 (*see* Note 24).
2. Calculating statistics. To make downstream analyses more efficient, ImmuneDB pre-calculates a number of statistics about clones and samples (*see* Note 25).

immunedb_clone_stats /share/configs/my_db.json
immunedb_sample_stats /share/configs/my_db.json
3. Create lineage trees for each clone. Optionally, lineage trees can be constructed for each clone. Like clonal inference, this process has many parameters, and the following is for general use and may need to be tweaked depending on sequencing depth, error rates, and the underlying biological samples:

immunedb_clone_trees /share/configs/my_db.json --min-seq-copies 2
  More details on clonal lineages can be found in Subheading 3.3 of the chapter “AIRR Community Guide to Repertoire Analysis.”

### 3.11 Analysis of Clone Numbers and Size Distributions

1. Sample clone count (*see* Note 26). One can do a quick “back-of-the-envelope” calculation to estimate the maximal number of expected unique IGH rearrangements in a bulk gDNA sequencing using the equation below [16] if the nanogram input is known:
$$\begin{array}{l} \text{Max}.\#\text{of}\;\text{rearrangements}\;\text{=}\;(\text{ng}\;\text{input})(\text{1000}\;\text{pg/ng})\\ \times (1.4\;\text{rearrangements}/\text{cell})/6.7\;\text{pg}/\text{cell}. \end{array}$$
  Or, equivalently, about 150 cells per nanogram of input DNA. These equations assume that 100% of the cells in the samples are the B or T cells of interest that there is quantitative recovery of all possible rearrangements and that each cell has an average of 1.4 rearrangements (due to some cells having more than one IGH or TRB rearrangement [17], *see* Note 27). Obtaining fewer or more clones than expected can reveal potential technical or analytical problems with the experiment or data analysis pipeline, respectively (*see* Note 28).
2. Clone size distribution. There are at least two size metrics which can be applied to each clone to generate distributions of estimated clone sizes. First, one can define the size of each clone as its number of sequence copies. This metric is particularly useful when looking at malignancies where nearly all copies reside in the same single clone (or a small number of clones). However, this approach can also be affected by PCR jackpots, sample-specific subset differences, or other inter-sample copy number variability (*see* Note 29). A second approach is to compute the number of unique sequences in each clone. This metric can be influenced by the level of SHM in the clone. It can also be affected by sequencing error, with larger numbers of unique sequences per clone arising in more deeply sequenced samples.
  - Histogram of top-ranked clones. As shown in Fig. 2a, one can plot the copy number fraction of the 20 clones in a sample that have the highest copy numbers. Investigating the top copy number clones in datasets can highlight expanded clones as compared to the overall repertoire, giving insight into a range of different biological processes (*see* Note 30). In healthy individuals, expanded B-cell clones in the peripheral blood generally have copy numbers within the same order of magnitude of non-expanded clones (*see* Note 31).
  - *D*_*x*_ index. One can compute the fraction of sequence copies that are occupied by the top *x* percent of clones in a sequencing library. *D*_*x*_ is the fraction of total copies occupied by the top *x* clones. A common value of *x* is 20 [10] which, when looking at copy number distribution, reveals if there are one or more dominating clones.
  - Clone rank plot. Unlike the top-ranked clone plot and *D*_*x*_ index, clone rank plots provide a snapshot of the clone size distribution in the entire repertoire. Clone rank plots achieve this by segregating clones by rank as shown in Fig. 2b. In such plots, each column represents a sample, or a pool of samples, and the height of each bar represents the proportion of copies in the given clonal range bracket. For example, in this example, the red bars show the proportion of sequence copies in the top ten ranked clones. In oligoclonal repertoires, both the *D*_*x*_ index and the clone rank plot, the top copy clones contain the majority of copies. In contrast, for polyclonal repertoires, range plots can provide a nuanced view of clonal abundance by stratifying clones into categories based on their copy number distributions.

### 3.12 Clonal Overlap Analysis

Determining how many samples or replicates are necessary to sufficiently reveal the clonal landscape of the underlying immune repertoire is challenging. Undersampling a repertoire can lead to underpowered analyses and false biological conclusions (e.g., claiming lack of overlap), whereas oversampling can be expensive and time-consuming.

1. Rarefaction analysis can provide insight into the level of sampling, providing a means of powering the clonal overlap analysis. Rarefaction stems from ecology where one wants to estimate the total number of species in a region by repeated sampling of organisms [18]. Species which occur in multiple independent samples are likely more abundant than those which only occur in a few samples. One can apply the same principles to the analysis of clones (*see* Note 32). This analysis is generally plotted as shown in Fig. 2c where the x-axis is the sample size (e.g., the number of clones sampled) and the y-axis is the diversity (e.g., the estimated total number of clones). Curves that plateau indicate that sampling more clones will likely not affect the total estimated number of clones in the underlying population. On the other hand, curves which do not flatten show that a larger sample size is required to reveal additional unknown clones.
2. Visualizing and quantifying clonal overlap. Clones which are found in multiple samples are of particular interest. The sizes of clones which are present at baseline and after treatment can reveal insights into the efficacy of treatment and a patient’s response to therapy. Clonal overlap can also be applied to samples which were acquired from different tissues, cell subsets, and other biologically relevant populations.
  - Clone definitions for the evaluation of clonal overlap. Most frequently used are clonal annotations or shared CDR3 amino acid sequences. In ImmuneDB, for example, clones are annotated with a unique clone ID that can be scanned across all of the samples in a given subject, allowing for the construction of clonal networks across all of the different samples in an individual. Alternatively, one can trace the consensus CDR3 amino acid sequence of each clone through samples to determine overlap.
  - The Jaccard index [19] is the cardinality of the intersection of two samples divided by the cardinality of the union of the same samples. Specifically, for two (potentially overlapping) sets of clones *A* and *B*, the Jaccard index *J* is calculated with
$$J=\frac{A\cap B}{A\cap B}$$
  - Cosine similarity. The cosine similarly also gives an indication of overlap between samples. However, unlike the Jaccard index, it takes into account clone size rather than only presence or absence in samples. For each of the two samples to compare, a one-dimensional vector is constructed, the values of which indicate the size of each clone in copies. The order of clone sizes must be the same for both samples. Specifically, given two vectors of clone sizes from two samples, *A* and *B*, the cosine similarity *S* is defined as
$$S=\frac{\sum_{i=1}^{n}A_{i}B_{i}}{\sqrt{\sum_{i=1}^{n}A_{i}}\sqrt{\sum_{i=1}^{n}B_{i}}}$$
  - Overlapping clones can be visualized in line plots (Fig. 2d) in which each column is a sample and each row is a clone (line). The lines can be heat mapped to indicate the abundance (e.g., copy number fraction) of a clone in a given sample. Line plots only show clones that overlap in two or more of the analyzed samples. To gain insight into the fraction of overlapping clones in each sample and their distribution, Venn diagrams (Fig. 2e) can be used. Venn diagrams show the numbers of overlapping and nonoverlapping clones but become difficult to visualize when four or more samples are being compared.
  - Clones can be further visualized in multidimensional datasets. The temporal relationship of sequences derived from the same clone can be further visualized as lineages which are rooted, directed graphs, showing the progression of mutations (Fig. 2f). This can be useful to analyze the changes within each clone across tissues, subsets, time-points, or other metadata of interest. There are multiple ways to infer lineages from a collection of clonally related sequences. Two common approaches are neighbor joining and maximum parsimony. Neighbor joining begins with every sequence being its own node and iteratively adds parent nodes between those which are most similar [20]. Maximum parsimony [21] takes as input the same sequences but instead attempts to construct a tree which requires the minimum number of total mutations. Both have positives and negatives. For example, neighbor joining can create trees which are not optimal (e.g., mutations occurring multiple times or incorrectly grouping clades), but it is computationally more efficient than maximum parsimony. Maximum parsimony, however, guarantees some properties of the tree such as minimizing its height, but is computational intractable to calculate for large clonal lineages.

## Figures and Tables

**Fig. 1:**
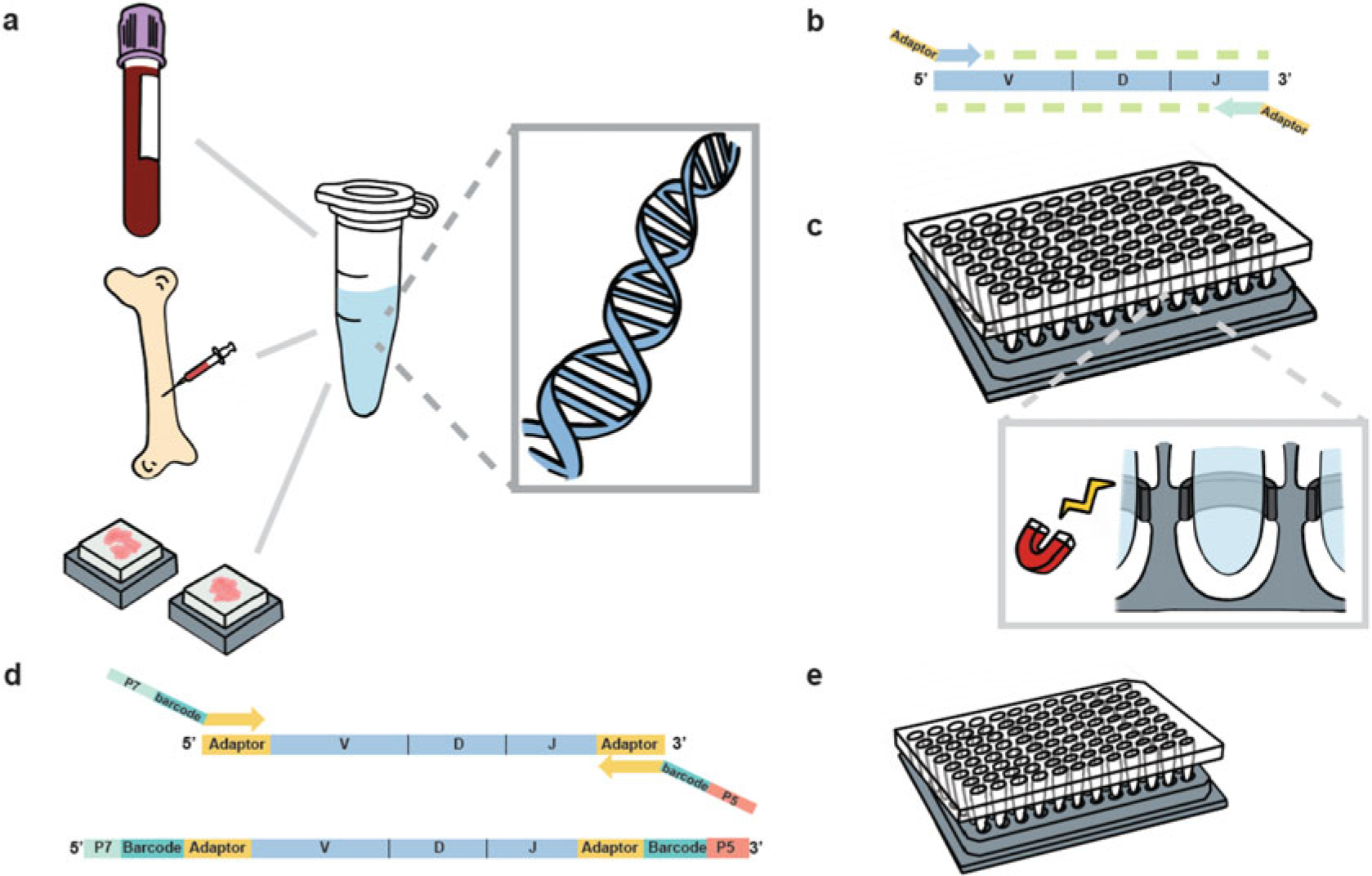
Workflow for IGH sequencing from bulk gDNA. (**a**) Starting from PBMCs, bone marrow aspirate, or formalin-fixed paraffin-embedded samples, gDNA is extracted from bulk populations. (**b**) Next, IGH gene rearrangements are amplified from gDNA using primer cocktails in FR1 and JH along with Illumina adapters. V = variable, D = diversity, and J = joining genes. (**c**) Amplicons from this first round of PCR are purified using AMPure beads and (**d**) subjected to second-round amplification using primers that include sample barcodes (see primers in Subheading 2.1 for DNA sequence information). (**e**) Sequencing libraries are subjected to further purification, size selection, quality control, and pooling prior to loading onto the sequencer

**Fig. 2:**
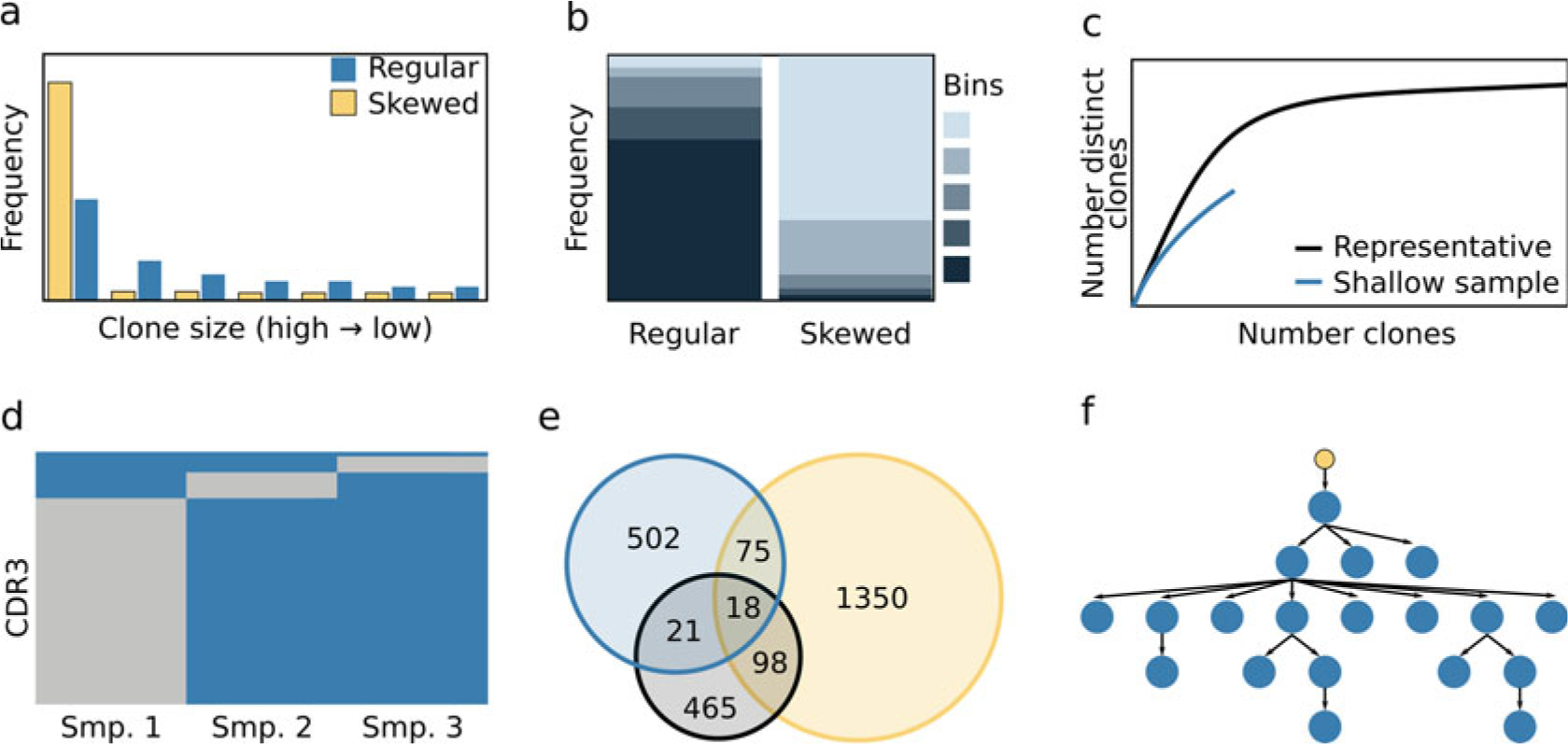
Clone visualization scheme. All plots are illustrative. (**a**) Top clone plot. An example plot showing the size of the top clones as measured by copy number in two samples, one shown in blue and one in yellow. Each set of columns represents the clone of a given rank, and the y-axis shows the copy number frequency as a fraction of the entire sample. (**b**) Clone rank plot. An example of a clone rank plot for two samples. Each bar represents a sample; each color represents the copy number fraction for a bin of clones of a given range of ranks (sizes) with lighter blue indicating higher-ranked (larger) clones and darker blue representing lower-ranked (smaller) clones. A generally darker sample indicates that the majority of clones are not expanded, and a lighter sample indicates a more oligoclonal repertoire. (**c**) Rarefaction curves. Illustrative rarefaction curves for two hypothetical samples showing sufficient and insufficient sampling. The x-axis indicates number of clones, and the y-axis indicates the measured number of total (unique) clones. Curves in which the number of distinct clones continues to increase as the number of sampled clones increases indicate potential undersampling (blue), whereas curves that begin to plateau (black) indicate the sampled clones are becoming more representative of the true underlying clonal population. (**d**) Clonal string plots visualizing the degree of clonal overlap between three samples. Each row represents a clone and each column a sample (smp). The presence of a clone in a given sample is indicated by blue and its absence by gray. Only clones that overlap in two or more samples are shown. (**e**) Venn diagram. Three different hypothetical samples (demarcated by the blue, yellow, and black circles) from the same individual. Numbers indicate clone counts that are found uniquely in one, two, or three of the samples. (**f**) Clonal lineage. An inferred hypothetical lineage of clonally related sequences. Each blue node represents a unique sequence, and the yellow node represents the nearest germline reference sequence. The edge length between two nodes indicates the total number of accumulated mutations from the parent sequence to the child sequence

## Footnotes

1. Sample barcodes can become associated with the wrong sample in the flow cell during sequencing (i.e., failure to accurately demultiplex the samples in a sequencing run), a phenomenon known as cross-clustering. For example, a very large clone in one sample can sometimes be found at very low copy number in an unrelated individual. Cross-clustering can also occur when the cluster density is too high. With dual indices, there is a second barcode that links a sample with an individual, providing a means of computationally resolving issues with cross-clustering [22].

2. All micropipet tips used in this protocol are SHARP^®^ Precision Barrier Tips (available from Thomas Scientific) that use low retention polymer technology. Some of the aerosol-resistant tips from other vendors trap liquids.

3. The recommended DNA inputs for different samples are up to 1 μg per reaction for formalin-fixed paraffin-embedded tissue (particularly if lymphopenic by histology), up to 400 ng/reaction for unsorted cells in which the cells of interest make up 5% or more of total nucleated cells or up to 100 ng/reaction for sorted lymphocytes per reaction. Lower input amounts can be used if less sample is available.

4. For fresh blood (collected within 1 h before starting the protocol), increase the incubation time to 8 min to ensure complete red blood cell lysis.

5. If the pellet does not break apart well, flick the bottom of the tube with your fingers.

6. If cell clumps are visible, incubate at 37 °C until the solution is homogeneous.

7. If RNA-free DNA is required, add 15 μl RNase A solution, and mix by inverting 25 times. Incubate for 15 min at 37 °C. Then incubate for 3 min on ice to quickly cool the sample for storage.

8. If the protein precipitation step does not form a tight pellet, incubate on ice for 5 min, and repeat the centrifugation at 4 °C.

9. If no DNA threads or clump is observed, add 5 μl of glycogen (20 mg/ml), and incubate the mix at −20 °C for 1 h.

10. The volumes of DNA and water can be adjusted based on DNA concentration and the experimental input. The volume of input DNA is recommended to be a minimum of 4 μl for pipetting accuracy.

13. Include up to 48 replicates in 1 96-well plate. Place the samples in every other well in rows and columns to reduce the risk of cross contamination. If fewer than 48 samples are used, spread the samples out on the plate as far apart as possible.

14. Ethidium bromide is a carcinogen. Wear appropriate personal protective equipment (lab coat, gloves, and eye protection), and discard the gel and disposables in the appropriate hazardous waste containers.

15. The beads need to be mixed well by vortexing briefly; then do a quick spin to bring down the leftover beads from the cap, and pipet up and down ten times right before mixing them with the amplicons.

16. All of these steps are performed in the post-PCR room.

17. The pooled library can also be stored at −20 °C until the day of the MiSeq run.

18. For survey-level sequencing, run at least two replicates (independent amplifications starting from gDNA). For deeper sequencing, run three or more replicates.

19. The reading of sample concentration from Qubit is in ng/μl and needs to be converted to nM using this formula: [Concentration by Qubit (ng/μl) × 10^6]/(660 × size of the amplicon in base pairs). The size of the IgH FW1 library amplicon is 510 bp.

20. 20 pM PhiX can be used for up to 3 weeks when aliquoted into LoBind tubes and stored at −20°C.

21. If one is using this method for the first time, a bioanalyzer analysis is highly recommended to evaluate the purity of the final library, and a KAPA quantification is recommended to compare with the Qubit concentration measurement. The method presented in this chapter uses Qubit for concentration measurement and uses 20 pM of the final library based on the Qubit calculation. Bioanalyzer and KAPA quantification may give different concentrations, and the optimal input library concentrations calculated based on these methods may differ.

22. Additional custom columns may be added for relevant metadata such as tissue, timepoint, etc., following the nomenclature conventions proposed by the AIRR Community [23]. For the updated AIRR-C nomenclature, please visit https://docs.airr-community.org/en/stable/datarep/metadata.html#repertoire-fields

23. The only technical limitations for database names are those documented in the MySQL requirements (https://dev.mysql.com/doc/refman/8.0/en/identifiers.html). In addition, we recommend that the names consist exclusively of lowercase Latin characters, integers, and underscores.

24. There are multiple built-in clonal inference methods including by edit distance and hierarchical clustering, both of which are highly customizable [24–27]. The –help command can be used to show additional clustering options.

25. After sequence alignment and clonal inference, it is useful to calculate high-level measures for each replicate (individual sequencing library), sample (pooled replicates), and subject. There are a number of different metrics that can indicate the quality of sequencing and also highlight potentially interesting biological phenomena, such as the number of total reads, valid reads (sequence copies), unique sequences, and clones. Copies, unique sequences, and clones can be heavily influenced by the input DNA amount and cell count. A low number of unique sequences as compared to copies can indicate PCR jackpots or oligoclonality of the underlying repertoire. A number of unique sequences that is similar to the total copies may indicate insufficient sequencing depth.

26. The clone count is influenced by the degree of clonal expansion, diversity of clones, and the amount (and purity) of the cell population of interest. The clone count is also influenced by how clonally related sequences are defined.

27. Typically cells with more than one IGH rearrangement have one productive and one nonproductive rearrangement as cells with two productive IGH [28, 29] or TRB rearrangements [30] are very infrequent. In contrast cells with two productive IGK [31] or TRA rearrangements [32] are more common.

28. If one obtains far fewer clones than expected, possible reasons include clonal expansion, low fraction of T cells or B cells in the sample, poor-quality template (such as an old FFPE sample [33]), technical problem with template amplification or sequencing such that only a few of the available rearrangements are being amplified, or a filtering procedure that results in an unacceptably large fraction of the data being removed or a clone collapsing procedure that groups unrelated sequences together into the same clones, under-calling the number of different clones. If, on the other hand, one obtains more clones than the predicted maximum number, there may be an issue with the computational pipeline in terms of how clones are defined. For example, if a very high level of sequence similarity is used on a sample enriched for memory B cells with high levels of SHM, clonally related sequences may be grouped falsely into separate clones.

29. If sampling a modest number of cells, in addition to spurious oligoclonality, the experiment may be more susceptible to artifacts such as PCR jackpots, in which one or a few templates “take over” the reaction, leading to misleading evidence of clonal expansion or dominance. The analysis of independently amplified biological replicates can provide important insights into clonal expansions (as discussed in greater detail in the method chapter “Quality Control: Chain Pairing Precision and Monitoring of Cross-Sample Contamination”). Clones which have a high copy number in one replicate but not others suggest a PCR jackpot or a highly oligoclonal sample with very few templates. Conversely, clones which reproducibly have high copy numbers may indeed be expanded.

30. Significant clonal expansions can be encountered in the setting of malignant or nonmalignant lymphoproliferative disorders or during acute immune responses. The degree of clonal expansion is influenced by the cell type under study (e.g., more expanded clones are encountered among memory populations than naive cells), the tissue (e.g., B cells make up a much larger fraction of total cells in the spleen than in the GI tract), and the level of sampling (sequencing libraries that contain fewer clones will have higher average numbers of copies per clone).

31. If one is surveying B cells in the peripheral blood, one can use the copy number fraction and fold-change over the next most frequent nondominant clone to report a significant clonal expansion. For example, one might use a cutoff of 5% of total sequence copies for an IGH rearrangement that is also at least threefold more frequent than the next most frequent nondominant rearrangement. In most individuals the top 20 most frequent clones in the blood typically occupy <2% of total sequence copies. The additional requirement of being threefold more frequent than the next most frequent nondominant rearrangement helps to limit false calls of significant expansions with oligoclonal samples (which could be due to poor sample quality, a low number of input cells due to B-cell lymphopenia, or other factors). The term nondominant is used in case there are expanded clones with more than one amplifiable IGH rearrangement, for example, one productive and one nonproductive IGH gene rearrangement in the same cell.

32. If multiple replicates are not available for the dataset of interest, one can also computationally resample the dataset, mimicking the effect of multiple replicates [34].
